# Salivary Hormones Leptin, Ghrelin, Glucagon, and Glucagon-Like Peptide 1 and Their Relation to Sweet Taste Perception in Diabetic Patients

**DOI:** 10.1155/2023/7559078

**Published:** 2023-05-15

**Authors:** Nada K. Al-Ghurayr, Ashjan M. Al-Mowalad, Ulfat M. Omar, Heba M. Ashi, Saad S. Al-Shehri, Abdelrahman A. AlShaikh, Shada M. AlHarbi, Hadeil M. Alsufiani

**Affiliations:** ^1^Biochemistry Department, Faculty of Sciences, King Abdulaziz University, Jeddah, Saudi Arabia; ^2^Princess Dr. Najla Bint Saud Al-Saud Center for Excellence Research in Biotechnology, King Abdulaziz University, Jeddah, Saudi Arabia; ^3^Department of Dental Public Health, Faculty of Dentistry, King Abdulaziz University, Jeddah, Saudi Arabia; ^4^Department of Clinical Laboratory Sciences, College of Applied Medical Sciences, Taif University, P.O. Box 11099, Taif 21944, Saudi Arabia; ^5^Department of Medicine, Faculty of Medicine, King Abdulaziz University, Jeddah, Saudi Arabia; ^6^Albawadi 1 Primary Health Care Center, King Fahad Hospital, Ministry of Health, Jeddah, Saudi Arabia; ^7^Experimental Biochemistry Unit, King Fahd Medical Research Center, King Abdulaziz University, Jeddah, Saudi Arabia

## Abstract

Diabetes mellitus (DM) is one of the most common diseases worldwide. DM may disrupt hormone regulation. Metabolic hormones, leptin, ghrelin, glucagon, and glucagon-like peptide 1, are produced by the salivary glands and taste cells. These salivary hormones are expressed at different levels in diabetic patients compared to control group and may cause differences in the perception of sweetness. This study is aimed at assessing the concentrations of salivary hormones leptin, ghrelin, glucagon, and GLP-1 and their correlations with sweet taste perception (including thresholds and preferences) in patients with DM. A total of 155 participants were divided into three groups: controlled DM, uncontrolled DM, and control groups. Saliva samples were collected to determine salivary hormone concentrations by ELISA kits. Varying sucrose concentrations (0.015, 0.03, 0.06, 0.12, 0.25, 0.5, and 1 mol/l) were used to assess sweetness thresholds and preferences. Results showed a significant increase in salivary leptin concentrations in the controlled DM and uncontrolled DM compared to the control group. In contrast, salivary ghrelin and GLP-1 concentrations were significantly lower in the uncontrolled DM group than in the control group. In general, HbA1c was positively correlated with salivary leptin concentrations and negatively correlated with salivary ghrelin concentrations. Additionally, in both the controlled and uncontrolled DM groups, salivary leptin was negatively correlated with the perception of sweetness. Salivary glucagon concentrations were negatively correlated with sweet taste preferences in both controlled and uncontrolled DM. In conclusion, the salivary hormones leptin, ghrelin, and GLP-1 are produced either higher or lower in patients with diabetes compared to the control group. In addition, salivary leptin and glucagon are inversely associated with sweet taste preference in diabetic patients.

## 1. Introduction

Diabetes mellitus (DM) is one of the most common diseases all over the world. DM is caused by an insulin deficiency or resistance and characterised by hyperglycaemia. The latest statistics reported that in 2021, more than 537 million individuals around the world have diabetes, and this number is expected to increase to 783 million by the year 2045 [1]. Saudi Arabia is found to be the second top country among the Middle East in regard to DM. This is due to the high prevalence (17.7%), where 1 in 6 adults is diagnosed with DM [2].

DM has severe complications such as atherosclerotic, heart failure, chronic kidney, retinopathy, and neuropathy diseases [3]. Moreover, it could cause disruption in hormone regulation [4]. The metabolic hormones leptin, ghrelin, glucagon, and glucagon-like peptide 1 (GLP-1) are produced by the salivary glands and taste cells, and the exact role of these salivary hormones is not completely understood [5]. Leptin, an adipocyte hormone, acts within the hypothalamus to control energy expenditure, food intake, and body weight [6]. The opposite of leptin is ghrelin, a 28-amino-acid polypeptide; it is an appetite hormone that signals hunger and, therefore, plays an important role in food intake [7]. Ghrelin also plays several important roles in oral function [8]. The third of the aforementioned hormones, glucagon, is produced by pancreatic *α*-cells of the islets of Langerhans. Its main function is to increase blood glucose concentrations. It is also expressed in a subset of taste cells of the tongue [9]. Finally, GLP-1 is an intestinal hormone that exerts profound effects on glycaemia and stimulates glucose-dependent insulin secretion [10]. In addition, GLP-1 and its receptor are involved in the transfer of sweet taste signaling to sweet-responsive cells, where they are coexpressed [11]. These salivary hormones are expressed at different concentrations in patients with diabetes compared to the control group and may cause differences in their perceptions of sweetness [5].

Taste perception, in general, is essential for living; it conveys information about food and influences food selection and dietary intake [12, 13]. The process of sweet taste perception begins with saliva, which dissolves sweet molecules and allows flavours to bind to taste cells [5, 14, 15]. Several studies have shown that patients with diabetes lack the proper response to the sensation of sweet taste [5, 16]. However, as far as we are aware, no studies have examined salivary hormones and their relationships with sweet taste perception in patients with diabetes. Therefore, the present study is aimed at assessing the concentrations of the salivary hormones leptin, ghrelin, glucagon, and GLP-1 and their correlations with sweet taste perception, including thresholds and preferences, in patients with controlled and uncontrolled DM compared to the control group.

## 2. Materials and Methods

### 2.1. Study Participants

**Figure 1:**
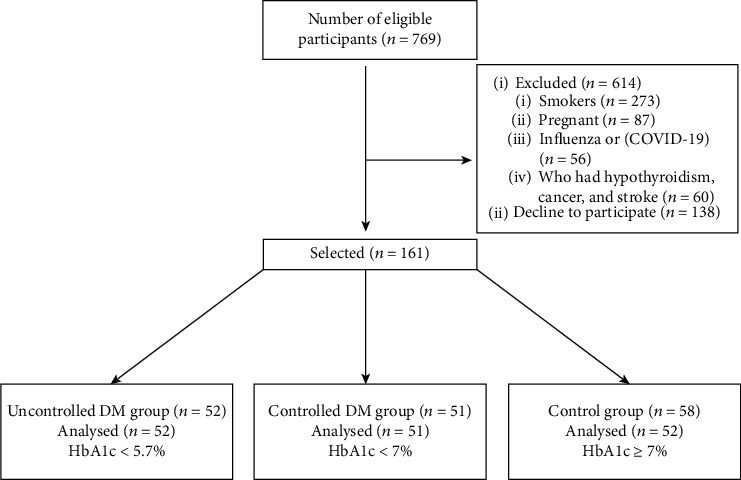
STROBE flow chart showing the flow of the participants throughout the study. *n*: number of patients; STROBE: Strengthening the Reporting of Observational Studies in Epidemiology.

A total of 155 male and female participants aged between 35 and 65 years were recruited from King Abdulaziz University Hospital and King Fahad Hospital Jeddah, Saudi Arabia, from January 2021 to December 2021. The total sample size was calculated based on a power analysis (using G^∗^Power software, version 3.1.9.7) that indicated a 95% chance of a 0.5 effect size between the tested groups at the 5% level (two-tailed). The participants were divided into three groups according to their glycated haemoglobin (HbA1c) concentrations. The participants in the control group (*n* = 52) had HbA1c concentrations of less than 5.7%, those in the controlled DM group (*n* = 52) had HbA1c concentrations of less than 7%, and those with uncontrolled DM (*n* = 51) had HbA1c concentrations of ≥7%. HbA1c data was obtained from participants' medical files, for diabetic within 3 months of test day and for control group within 6 months of test day. Patients who had hypothyroidism, stroke, and cancer or were pregnant and smokers, used antibiotics, had oral lesions, had influenza or COVID-19, had been exposed to radiation in the head or neck regions, or were under 35 years or above 65 years of age were excluded (Figure 1). The study was approved by the ethical committees of the Faculty of Medicine, King Abdulaziz University (ref. no. HA-02-J-008) and King Fahad Hospital (ref. no. 679-20). All participants provided written informed consent.

### 2.2. Anthropometric Measurements

Before each participant entered the clinic, a nurse measured their weight and height using a Medical Electronic Body Height Weight Scale in the screening room. These measurements were used to calculate each participant's body mass index (BMI). In addition, each participant's waist and hip circumferences were measured using a fabric measuring tape, and these measurements were used to calculate each participant's waist-to-hip ratio (WHR).

### 2.3. Salivary Hormone Measurements

A saliva sample was collected from each participant in an odourless room during morning (from 8 AM to 11 AM). Each participant fasted for one hour prior to the sample collection. The participants rinsed their mouths with distilled water to remove any excess food. Next, the head was tilted forward for some minutes, and a 2 ml of saliva was collected (without stimulators) in a sterilised tube from the anterior of the mouth and placed directly on ice. Then, the saliva samples were centrifuged at 1000 × *g* for 20 minutes. The supernatant was then collected and stored at -80°C until the analysis. The salivary hormone concentrations were determined using sandwich enzyme-linked immunosorbent assay kits (Bioassay Technology Laboratory, Saliva-Human ELISA Kit), according to the manufacturer's protocol. Human Leptin ELISA kit (E1559Hu), Human Ghrelin ELISA kit (E1552Hu), Human Glucagon ELISA kit (E1023Hu), and Human Glucagon-like Peptide-1 ELISA kit (E0022Hu) were used to determine leptin, total ghrelin, glucagon, and GLP-1 concentrations, respectively.

### 2.4. Assessment of Sweet Taste Perception and Habitual Sweet Intake

Each participant's sweet taste perception including sweet taste threshold and sweet taste preference was assessed. The sweetness threshold (also known as sucrose recognition threshold) was determined in the following manner: the participant was offered different concentrations of a sucrose solution (0.015, 0.03, 0.06, 0.12, 0.25, 0.5, and 1 mol/l), in ascending order, until they identified a sweet taste [17]. After each concentration, the participant rinsed their mouth with distilled water. The sweet taste preference was measured in the same manner (the participant was offered different concentrations of a sucrose solution (0.015, 0.03, 0.06, 0.12, 0.25, 0.5, and 1 mol/l), in ascending order [17]), until the participant identified their preferred sucrose solution. Each participant also completed a short questionnaire to assess their habitual sweet intake. The questionnaire consists of questions regarding the preference for sweet foods over other foods, the patients' consumption of sweet foods, whether they always crave sweets, and whether they believe that sweet foods affect their health.

### 2.5. Statistical Analysis

One-way analysis of variance (ANOVA) followed by Dunnett's multiple comparison test was used to assess differences in the concentrations of leptin, ghrelin, glucagon, and GLP-1 between the three groups (control, controlled DM, and uncontrolled DM). In addition, this test was also used to determine the differences in the sweetness thresholds and preferences between the three groups. The relationships between each salivary hormone and HbA1c, sweet taste threshold, and sweet taste preference were tested using the Spearman rank correlations. All statistical analyses were performed using GraphPad Prism software (version 9.3.1, USA, Biomatters, Ltd. NZ and GSL Biotech, USA). A *P* value of < 0.05 was considered statistically significant.

## 3. Results

### 3.1. Demographic Characteristics of the Study Participants

This study included a total of 155 participants; their demographic characteristics are shown in Table 1. Fifty-nine percent of the participants were women, while 41% were men. The mean age was 52.81 ± 7.64 years. The mean BMI was 30.71 ± 5.65 (kg/m^2^). The mean WHR was 0.86 ± 0.22, and the mean HbA1c was 6.73 ± 1.70%. The participants in the controlled and uncontrolled DM groups had diabetes for a mean duration of 10.04 ± 8.64 and 13.23 ± 7.41 (years), respectively. In the control group, 54% of the participants has been diagnosed with hypertension. This percent was slightly higher in controlled DM and uncontrolled DM (65 and 58%, respectively). With regard to sweet intake, 40%, 53%, and 63% of the participants in the control group, controlled DM, and uncontrolled DM did not prefer sweet food over other foods, respectively. Although almost half of the participants in each group always crave sweets, more than half of them did not consume much sweet food. Moreover, 82% of the participants in the control group believed that sweet foods affect their health. Similar belief was also found in diabetic patients.

### 3.2. Salivary Hormones Leptin, Ghrelin, Glucagon, and GLP-1 Concentrations

**Figure 2:**
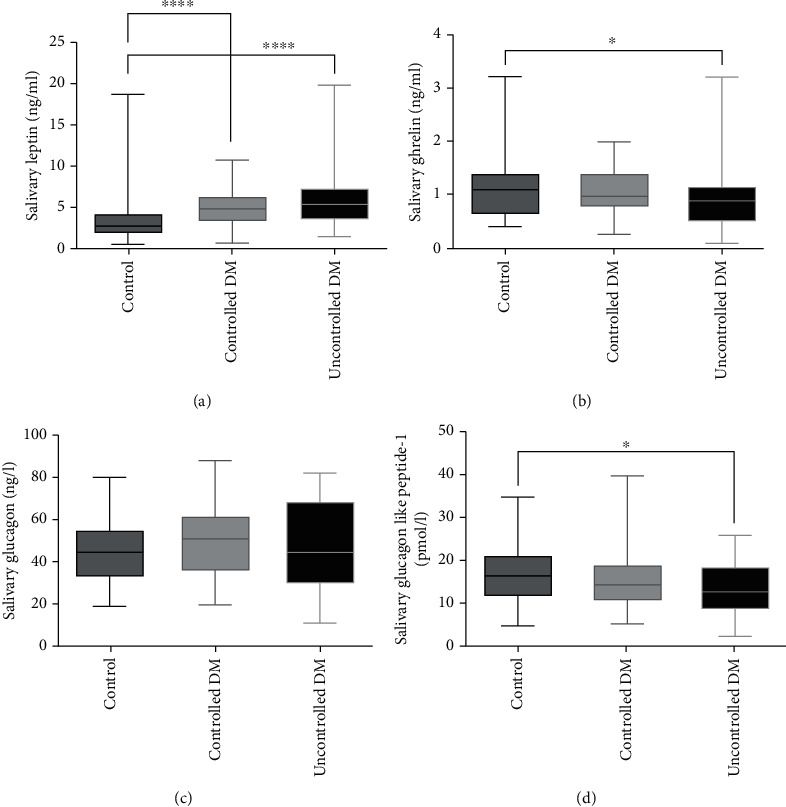
Mean salivary hormones leptin (ng/ml) (a), ghrelin (ng/ml) (b), glucagon (ng/l) (c), and GLP-1 (pmol/l) (d) concentrations in the three groups (control, controlled DM, and uncontrolled DM). The mean concentrations of salivary leptin differed significantly between the control group and both the controlled and uncontrolled DM groups (a, ^∗∗∗∗^). The salivary ghrelin and GLP-1 concentrations differed significantly between the control group and the group with uncontrolled DM (b, d, ^∗^). The salivary glucagon concentrations differed between all 3 groups, but these differences were not significant (c). ^∗^*P* < 0.05. ^∗∗∗∗^*P* < 0.0001. DM: diabetes mellitus; GLP-1: glucagon-like peptide-1.

The mean concentrations of leptin, ghrelin, glucagon, and GLP-1 in each of the three groups are shown in Figure 2. The results showed that salivary leptin was significantly higher in the groups with controlled and uncontrolled DM than in the control group (Figure 2(a)). In contrast, salivary ghrelin concentrations were significantly lower in the uncontrolled DM group than in the control group. In addition, the salivary ghrelin concentrations were also lower in the controlled DM group than in the control group, but this difference was not significant (Figure 2(b)). With regard to salivary glucagon, there were nonsignificant differences between all three groups (Figure 2(c)). Finally, the GLP-1 concentrations were significantly lower in the uncontrolled DM group than in the control group. GLP-1 concentrations were also lower in the controlled DM group than in the control group, but this difference was not significant (Figure 2(d)).

### 3.3. Sweet Taste Thresholds and Preferences

**Figure 3:**
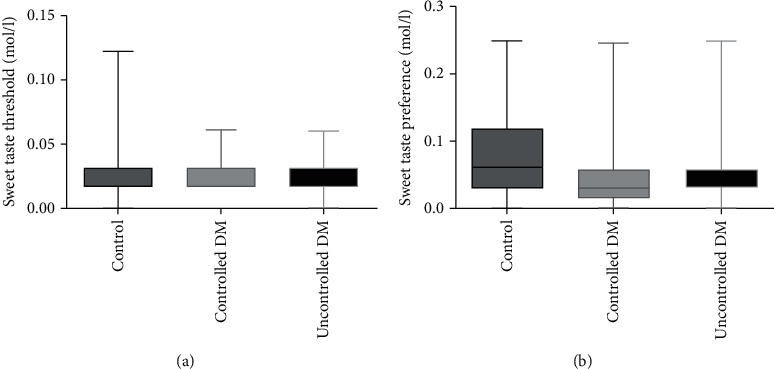
Differences in the perception of sweetness, including threshold and preference, between all three groups. The mean sweet taste thresholds (a) and sweet taste preferences (b) of the study participants differed nonsignificantly between all three groups. DM: diabetes mellitus.

The differences in the sweet taste thresholds and preferences between the three groups (control, controlled DM, and uncontrolled DM) are shown in Figure 3. There were nonsignificant differences in the sweet taste thresholds (Figure 3(a)) and the sweet taste preferences between all groups (Figure 3(b)).

### 3.4. The Relationships between Salivary Hormones and HbA1c, Sweet Taste Thresholds, and Sweet Taste Preferences

The correlation between salivary leptin, ghrelin, glucagon, and GLP-1 concentrations and HbA1c, sweet taste thresholds, and sweet taste preferences for all three groups are shown in Table 2. In general, HbA1c was positively correlated with salivary leptin concentrations and negatively correlated with salivary ghrelin concentrations. In the group with controlled DM, salivary leptin was negatively correlated with sweet taste threshold and sweet taste preference. In the group with uncontrolled DM, salivary leptin was negatively correlated with sweet taste preference only. In the groups with controlled and uncontrolled DM, salivary glucagon was negatively correlated with sweet taste preference. In contrast, no correlation was found between salivary hormones ghrelin and GLP-1 in the control, controlled DM, and uncontrolled DM groups and sweet taste threshold and sweet taste preference.

## 4. Discussion

The purpose of this study was to assess the concentrations of the salivary hormones leptin, ghrelin, glucagon, and GLP-1 and their relationships with the perceptions of sweetness in individuals with controlled and uncontrolled DM as compared with control individuals.

### 4.1. Salivary Hormones Leptin, Ghrelin, Glucagon, and GLP-1

The results of our study demonstrated that salivary leptin concentrations were higher in the uncontrolled and controlled DM groups than in the control group. In addition, there was a strong positive correlation between salivary leptin and HbA1c in all groups. This finding is consistent with the finding of a previous study which reported that salivary leptin was twofold higher in DM patients compared to healthy controls. Moreover, they found a positive significant relationship between salivary leptin and insulin and some inflammatory markers. These correlations may indicate that the increment in salivary leptin observed in our study could be due to the situation of insulin resistance and inflammation occurring in DM, since leptin can act as proinflammatory cytokine [18]. In contrast, salivary ghrelin concentrations were lower in the uncontrolled DM group than in the control group. Furthermore, in all groups, salivary ghrelin was negatively correlated with HbA1c. These findings are consistent with the results from a previous study which showed a decrease in salivary ghrelin in obese diabetic patients compared to healthy group. The authors attributed this change to diabetes not obesity, and the explanation of this decrease is not clear until now [19]. We found no differences in salivary glucagon concentrations between the three groups in this study. To our knowledge, no previous studies have been performed regarding salivary glucagon concentrations in this context. The concentrations of GLP-1 were lower in the uncontrolled DM group than in the control group. Similarly, Young et al. reported an inverse correlation between gut GLP-1 and blood glucose concentrations in diabetic patients. Blood glucose concentrations were inversely correlated with the expression of the taste receptors (taste 1 receptor member 2 (T1R2) and taste 1 receptor member 3 (T1R3)) located in the upper gastrointestinal mucosa of diabetic patients. These taste molecules were found to be linked to GLP-1 release; a downregulation could impair GLP-1 secretion [20]. Since the expression of these taste molecules is the same as in the tongue, we could indicate that the lower concentration of salivary GLP-1 in our patients may be due to the lower expression of T1R2 and T1R3 as a consequence of high blood concentrations.

### 4.2. Sweet Taste Thresholds and Preferences

In our study, the three groups showed no differences with respect to sweet taste thresholds and preferences. Similarly, previous study conducted by Wasalathanthri et al. reported no difference in sweet recognition threshold between patients with normal glycaemic levels and those with diabetes [21]. Furthermore, a recent cross-sectional study conducted by Trius-Soler et al. did not found relationship between sweet taste thresholds and diabetes antecedents in a healthy college-aged cohort [22]. In contrast with our results, a meta-analysis done by Trius-Soler et al. which include a great number of studies and patients found a significant increase in the sucrose recognition threshold in type 2 diabetes mellitus. This difference was due to environmental, physiological, and genetic factors [23]. Also, another study by Yu et al. reported a decrease in sweetness preference in diabetic patients. The authors suggested that this decrease may be due to the psychological aversion to sweet foods which in turn cause abnormal sucrose perception and then cause alteration in sucrose preference [24]. This inconsistency may be due to the similar habitual sweet intake between the control and diabetic patients observed in this study which lead to similar sweet taste threshold and sweet taste preference.

### 4.3. The Relationships between Salivary Hormones and Sweet Taste Thresholds and Preferences

Our results indicated that in the controlled DM group, salivary leptin was negatively correlated with sweet taste threshold and sweet taste preference; similar findings were also seen for the uncontrolled DM group with respect to sweet taste preference. These results are similar with the previous results conducted on mice which showed a strong correlation between leptin and sweet taste perception. Leptin suppresses sweet taste via leptin receptor (Ob-Rb) and K_ATP_ channels that are expressed in taste cells which in turn suppress their responses to sweet compounds [25]. As for salivary glucagon, our results showed in both the controlled and uncontrolled DM groups a negative correlation between the hormone and sweetness preferences. To our knowledge, no previous studies have been conducted with respect to salivary glucagon in this context. In our study, no correlations between salivary ghrelin and sweet taste threshold and sweet taste preference were found. This contrasts a previous study which reported ghrelin effect on sweet taste sensitivity, and this inconsistency may be due to the source of their sample (blood rather than saliva) [26]. In addition to ghrelin, salivary GLP-1 was not correlated to sweet taste threshold nor perception. Contrary to the findings of our study, Takai et al. reported that GLP-1 decreased nerve responses to the sweet stimuli for glucose, maltose, fructose, and saccharin. The contrast with our study may be due to the different sugars other than sucrose used in their study [11], while sucrose is the sweetest compared with other sugars [27].

## 5. Conclusions and Future Research

To the best of our knowledge, this study is the first to report the associations between salivary leptin, ghrelin, glucagon, and GLP-1 concentrations and sweet taste perceptions, including sweet taste thresholds and preferences, in individuals with controlled DM, those with uncontrolled DM, and those without DM. The salivary hormones leptin, ghrelin, and GLP-1 are either higher or lower in patients with diabetes compared to the control group. In addition, salivary leptin and glucagon are inversely associated with sweet taste preference in diabetic patients. However, a number of limitations need to be considered, for instance, measurement of the expression of taste receptors in the tongue. Thus, future research should be done to investigate the mechanism of action of these salivary hormones on sweet taste and the expression of taste receptors.

## Figures and Tables

**Table 1:** Demographic characteristics of the study participants.

	Control group (*n* = 52)	Controlled DM group (*n* = 52)	Uncontrolled DM group (*n* = 51)	All groups (*N* = 155)
Gender (*n*, %)				
Female	32 (61.5)	33 (63.5)	26 (51)	91 (59)
Male	20 (38.5)	19 (36.5)	25 (49)	64 (41)
Age (years)	51.06 ± 8.24	53.42 ± 7.60	53.96 ± 6.81	52.81 ± 7.64
BMI (kg/m^2^)	30.74 ± 5.85	30.35 ± 6.18	31.13 ± 4.91	30.71 ± 5.65
WHR	0.84 ± 0.20	0.84 ± 0.25	0.91 ± 0.19	0.86 ± 0.22
HbA1c (%)	5.29 ± 0.26	6.18 ± 0.53	8.75 ± 1.39	6.73 ± 1.70
Duration of diabetes (years)	—	10.04 ± 8.64	13.23 ± 7.41	11.65 ± 8.17
Diagnosed with other disease (*n*, %)				
Yes	28 (54)	33 (65)	30 (58)	91 (59)
No	24 (46)	18 (35)	22 (42)	64 (41)
Prefer sweet foods over other foods (*n*, %)				
Yes	24 (46)	21 (41)	18 (35)	63 (41)
No	21 (40)	27 (53)	33 (63)	81 (52)
Sometimes	7 (13)	3 (6)	1 (2)	11 (7)
High consumer of sweets (*n*, %)				
Yes	10 (19)	8 (16)	5 (10)	23 (15)
No	30 (58)	37 (73)	35 (67)	102 (66)
Sometimes	12 (23)	6 (12)	12 (23)	30 (19)
Always crave sweets (*n*, %)				
Yes	26 (50)	27 (53)	23 (44)	76 (49)
No	12 (23)	12 (24)	16 (31)	40 (26)
Sometimes	14 (27)	12 (24)	13 (25)	39 (25)
Believe that sweet foods affect their health (*n*, %)				
Yes	40 (82)	49 (96)	45 (88)	134 (89)
No	9 (18)	2 (4)	6 (12)	17 (11)

Data are presented as means ± standard deviations (SD) or number (%). DM = diabetes mellitus; BMI = body mass index; WHR = waist-to-hip ratio; HbA1c = glycated haemoglobin.

**Table 2:** Spearman correlations coefficients (*r*) between salivary leptin, ghrelin, glucagon, and GLP-1 concentrations and HbA1c concentrations, sweet taste thresholds, and sweet taste preferences in all three groups.

Salivary hormone	HbA1c	Sweet taste threshold	Sweet taste preference
Leptin			
Control	0.06361	0.05120	-0.02597
Controlled DM	-0.08021	-0.3504^∗^	-0.3445^∗^
Uncontrolled DM	0.09704	0.01583	-0.3377^∗^
Leptin in all groups	0.3620^∗∗∗∗^	-0.09466	-0.07626
Ghrelin			
Control	-0.05115	0.09236	-0.05674
Controlled DM	0.04879	-0.2690	-0.2445
Uncontrolled DM	0.05228	0.07383	-0.2499
Ghrelin in all groups	-0.1686^∗^	-0.006995	0.01981
Glucagon			
Control	-0.06395	0.1668	0.1165
Controlled DM	-0.01762	-0.1791	-0.4393^∗∗^
Uncontrolled DM	0.1439	-0.1061	-0.4068^∗∗^
Glucagon in all groups	0.03269	-0.06499	0.1182
Glucagon-like peptide 1			
Control	-0.007643	0.08940	-0.009461
Controlled DM	0.1275	-0.1069	0.03959
Uncontrolled DM	0.1255	-0.05740	-0.08888
Glucagon-like peptide 1 in all groups	-0.1077	-0.04525	0.03625

^∗^ *P* < 0.05. ^∗∗^*P* < 0.01. ^∗∗∗∗^*P* < 0.0001. DM: diabetes mellitus; HbA1c: glycated haemoglobin.

## Data Availability

The data that support the findings of this study are available from the corresponding author upon reasonable request.

## Abbreviations

DM: Diabetes mellitus
GLP-1: Glucagon like peptide-1
HbA1c: Glycated haemoglobin
STROBE: Strengthening the Reporting of Observational Studies in Epidemiology
BMI: Body mass index
WHR: Waist-to-hip ratio
ELISA: Enzyme-linked immunosorbent assay
ANOVA: One-way analysis of variance
SEM: Standard error of the mean.
